# A comparison of novel silicone plate vs. auricular cartilage in upper eyelid reconstruction following excision of malignant tumor

**DOI:** 10.22336/rjo.2023.27

**Published:** 2023

**Authors:** Salil Kumar Mandal, Anwesha Maitra, Oishik Sarkar, Paulomi Roy, Mousree Gayen, Anamika Paul

**Affiliations:** * Department of Orbit, Oculoplasty and Reconstructive Surgery, Regional Institute of Ophthalmology, Medical College and Hospital, Kolkata, India

**Keywords:** silicone plate, auricular cartilage, reconstruction, eyelid, tumor

## Abstract

**Purpose:** To compare surgical and functional outcomes, safety, efficacy and cost of silicone plate vs. autogenous auricular cartilage (AAC) as alternate material to tarsal plate for upper eyelid reconstruction after excision of malignant tumor.

**Methods:** A prospective, comparative, interventional study of over 3 years was conducted on two groups of twenty patients each. All the patients had undergone the Modified Cutler Beard procedure with AAC being used as tarsal substitute in one group and a novel silicone plate in the other. Post-operative MRD 1, LPS action, Central Lid Thickness, and Lid contour were recorded at one week, one month and six months follow-up.

**Results:** The pre-operative MRD 1 in the silicone plate and AAC group was -2.95 ± 1.19 mm and -3.05 ± 1(1).05 mm, post-operative in the silicone plate group 3.8 ± 0.4 mm, and in the AAC group, 3.8 ± 0.41 mm. The pre-operative LPS action in the silicone plate and AAC group was 1.2 ± 1.1 mm and 1.0 ± 0.9 mm and post-operative it was 13.8 ± 0.4 mm for the silicone plate group and 13.7 ± 0.4 mm for the AAC group. The post-operative lid thickness for the silicone plate group was 4.4 ± 0.17 mm and for the AAC group it was 4.4 ± 0.08 mm.

**Conclusion:** The cosmetic outcome in terms of lid contour maintenance is better in the silicone plate group, in which it markedly reduces the surgical time, provides earlier rehabilitation, and eliminates disease transmission. Harvesting of AAC is a skillful and time-consuming procedure and adds to the post-operative morbidity due to the presence of a second surgical site. The low manufacturing cost of silicone plate as opposed to other allogenic and synthetic tarsal substitutes makes it readily available to resource limited populations. The silicone plate is reckoned to become the material of choice as tarsal substitute in the future.

**Abbreviations: **AAC = Autogenous auricular cartilage, MRD-1 = Margin reflex distance-1, LPS = levator palpebrae superioris, PFH = palpebral fissure height

## Introduction

The eyelids are flexible shutter-like structures that protect the surface of the eyeball. The eyelids also perform important functions, such as even maintenance of the tear film over the cornea, drainage of the tears by conjunctival and lacrimal pump action and regulation of the amount of light entering the eye. The eyelids and eyebrows contribute to the facial features of an individual and by their position relay useful information about the state of wakefulness and attention. They close every six seconds by reflex action [**1**]. Eyelid defects are caused by surgical resection of tumors [**2**-**4**], traumatic injuries or congenital anomalies. The Cutler Beard procedure was a crucial advancement with respect to repair of large upper eyelid defects [**5**-**8**]. However, in the absence of any tarsal substitute, complications like upper lid entropion [**9**], dermatochalasis and lid shrinkage began to arise with the conventional Cutler Beard procedure.

Different materials have been used as tarsal plate substitutes in several studies conducted. In 2016, Mandal SK et al. achieved a satisfactory outcome with autogenous auricular cartilage as tarsal substitute in Modified Cutler-Beard procedure [**10**]. However, a second surgical site, increased surgical time and uneven thickness of harvested cartilage were some of its disadvantages. Despite the excellent post-operative tissue acceptance and tissue adaptability, the newly created upper lid was thicker and not cosmetically satisfactory. In 2021, Mandal SK et al. used a silicone plate fashioned from a commercially available scleral buckle as tarsal substitute [**11**]. This silicone plate was found to overcome all the disadvantages encountered with the tarsal substitutes previously used. In this study, we have attempted to compare surgical outcomes, functionality and cosmesis of silicone plate with auricular cartilage as a tarsal plate substitute in upper eyelid reconstruction. 

## Aims and objectives 

To evaluate the surgical outcomes and efficacy of silicone plate vs. auricular cartilage for tarsal plate replacement in the repair of 60-100% eyelid defects after excision of malignant tumor; to appraise the cost and safety of using silicone plate and auricular cartilage; to assess the recurrence of malignant tumor; to assess the functional and aesthetic outcomes.

## Methods

A prospective, comparative, interventional study over a period of three years was conducted on 40 patients divided into two groups: auricular cartilage group: 20 patients; silicone plate group: 20 patients. The inclusion criteria were: all upper eyelid malignant tumors with upper eyelid created defect of 60-100% after the removal of the tumor. The exclusion criteria were: involvement of local lymph nodes, distant metastasis in liver, lung or brain, associated lower eyelid involvement, gross corneal infiltration, and intra-orbital extension.

## Pre-operative workup 

**Fig. 1:**
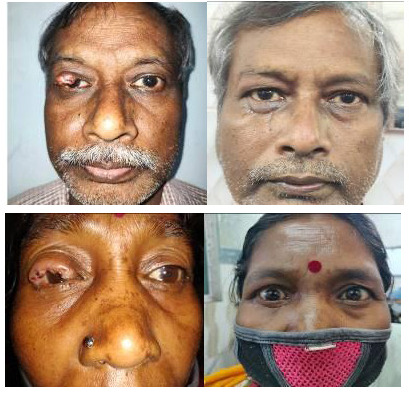
Pre-operative and post-operative image of silicone plate group

**Fig. 2:**
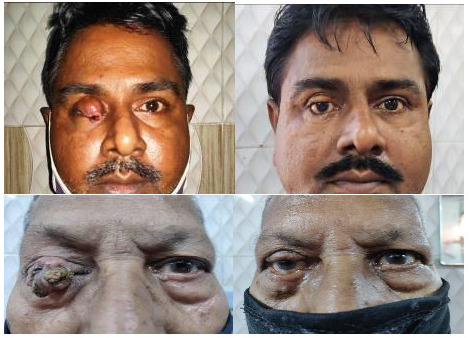
Pre- and post-operative image of auricular cartilage group

Pre-operatively, Marginal Reflex Distance - 1, Levator Palpebrae Superioris action, Palpebral Fissure Height and percentage of the lid involvement by the tumor were noted. All the patients have undergone a preoperative confirmation of the diagnosis of upper lid malignant tumor by Fine Needle Aspiration Cytology or incisional biopsy. MRI brain and orbit were done to exclude the spread of the tumor beyond the eyelid, i.e., intra-orbital spread. Clinical photographs were taken (**Fig. 1**, **2**, **Table 1**, **2**).

**Table 1:** Silicone plate group shows age, sex, percent of lid defect, operating time, pre- and post-operative MRD-1, pre- and post-operative LPS action, pre- and post-operative PFH, pre- and post-operative lid thickness

Age (years)	Gender	Tumor Lid Involvement (%)	Intra-operative lid defect (%)	Operating time of stage I (mins)	Pre-operative MRD 1 (mm)	Post-operative MRD 1 (mm)			Pre-operative LPS action (mm)	Post-operative LPS action (mm)			Pre-operative Palpebral Fissure Height (mm)	Post-operative Palpebral Fissure Height (mm)			Post-operative lid thickness (mm)		
						At 1 week	At 1 month	At 6 months		At 1 week	At 1 month	At 6 months		At 1 week	At 1 month	At 6 months	At 1 week	At 1 month	At 6 months
55	Male	56	70	45	-3	2	3	4	1	12	13	14	2	7	8	9	6.5	5.5	4.3
51	Female	80	93	50	-4	2	2	4	0	12	12	14	1	7	7	9	6.5	5	4.5
62	Female	76	90	40	-4	1	2	3	0	11	13	14	1	6	7	8	7	6.5	4.5
78	Female	56	70	55	-2	2	3	4	2	12	14	14	3	7	8	9	6	5	4.2
58	Female	70	83	45	-4	2	3	3	1	11	12	14	1	7	8	8	6.5	6	4.4
60	Female	86	100	45	-4	1	2	4	0	11	13	14	1	6	7	9	6.5	6	4.6
48	Female	86	100	50	-5	1	2	4	0	11	11	13	0	6	7	9	7.5	6.5	4.4
72	Male	63	76	50	-1	2	3	4	3	12	13	14	4	7	8	9	6	5.5	4.2
45	Male	76	90	45	-3	1	2	4	1	11	12	13	2	6	7	9	6.5	5.5	4
44	Female	80	93	55	-3	1	2	4	1	11	12	14	2	6	7	9	6	5.5	4.5
64	Male	80	100	40	-2	2	3	4	2	12	12	14	3	7	8	9	6	5.5	4.4
46	Female	80	90	45	-4	0	2	3	0	10	11	13	1	5	7	8	6.5	6	4.5
53	Male	63	76	50	-1	2	3	4	3	12	12	14	4	7	8	9	6.5	5.5	4.3
54	Female	66	76	45	-2	2	3	4	2	11	12	14	3	7	8	9	8	8	4.8
62	Male	76	90	65	-4	1	2	3	0	10	12	14	1	6	7	8	7	6	4.5
44	Female	76	93	50	-4	1	2	4	0	11	11	13	1	6	7	9	6.5	6	4.5
67	Female	70	83	45	-3	2	3	4	1	11	12	14	2	7	8	9	6.5	5.5	4.5
50	Male	66	83	40	-3	2	3	4	1	11	11	14	2	7	8	9	7	6.5	4.5
42	Male	66	80	45	-1	2	3	4	4	12	13	14	4	7	8	9	6.5	5.5	4.2
56	Male	60	73	45	-2	1	3	4	2	11	14	14	3	6	8	9	6.5	6	4.3

**Table 2:** Autogenous auricular cartilage group showed age, sex, percent of the lid defect, operating time, pre- and post-operative MRD-1, pre- and post-operative LPS action, pre- and post-operative PFH, pre- and post-operative lid thickness

Age (years)	Gender	Tumor Lid Involvement (%)	Intra-operative lid defect (%)	Operating time of stage I (mins)	Pre-operative MRD 1 (mm)	Post-operative MRD 1 (mm)			Pre-operative LPS action (mm)	Post-operative LPS action (mm)			Pre-operative Palpebral Fissure Height (mm)	Post-operative Palpebral Fissure Height (mm)			Post-operative lid thickness (mm)		
						At 1 week	At 1 month	At 6 months		At 1 week	At 1 month	At 6 months		At 1 week	At 1 month	At 6 months	At 1 week	At 1 month	At 6 months
49	Female	66	76	155	-2	1	2	4	2	9	11	14	3	6	7	9	7	6.7	4.6
56	Female	86	100	160	-5	0	1	3	0	7	8	13	0	5	6	8	8	7.5	4.5
55	Male	86	100	160	-4	0	1	4	0	7	8	13	1	5	6	9	7.5	7	4.5
63	Female	86	100	165	-4	1	2	4	0	6	8	14	1	6	7	9	7.2	7	4.4
76	Female	60	73	175	-2	1	2	4	3	7	8	14	3	6	7	9	7.4	7	4.6
64	Female	63	76	170	-2	1	2	4	1	8	9	14	3	6	7	9	7.5	7	4.5
69	Male	83	96	185	-4	1	1	4	1	7	8	13	1	6	7	9	7.6	7.1	4.5
46	Male	86	100	180	-4	0	1	3	0	7	8	14	1	5	6	8	7.5	7.4	4.5
66	Female	66	80	190	-2	1	2	4	2	8	8	14	3	6	7	9	7	6.8	4.4
61	Male	73	86	175	-2	1	2	4	1	7	8	14	3	6	7	9	7.5	6.5	4.5
59	Male	76	90	180	-3	1	1	4	1	7	8	14	2	6	7	9	7.5	6.5	4.5
51	Female	73	78	165	-3	1	2	4	1	7	8	13	2	6	7	9	7.2	7	4.4
51	Male	86	100	180	-4	0	1	4	0	6	8	13	1	5	6	9	7	6.5	4.5
55	Female	86	100	180	-4	1	2	3	0	7	8	14	1	6	7	8	7.4	6.8	4.6
48	Female	70	83	175	-2	1	2	4	2	8	9	14	3	6	7	9	8	7.6	4.6
52	Male	73	86	160	-3	1	2	4	1	8	9	14	2	6	7	9	7.5	6.5	4.5
49	Female	70	70	155	-3	1	1.5	4	1	7	8	14	2	6	6.5	9	7.5	6.5	4.6
58	Female	86	100	165	-4	1	2	4	0	6	7	13	1	6	7	9	7.2	6.8	4.3
67	Male	73	86	145	-3	1	2	3	1	7	8	14	2	6	7	8	7.5	6.7	4.5
72	Female	63	76	150	-1	1	1.5	4	3	8	8	14	4	6	6.5	9	7.4	6.5	4.4

## Surgical procedure

In this study, an experienced surgeon has performed all the operations in the same setting, under general anaesthesia. In the first stage of the Modified Cutler Beard procedure, the incision was marked first 4 mm away from the tumor margin. The tumor was excised completely, leaving a rectangular defect in the upper lid. Intraoperative frozen section control was used to confirm tumor free margin of the excised tissue in all cases. In the lower eye lid, 4-6 mm below its upper border, a horizontal incision was made followed by two vertical incisions, and were joined to create a rectangular flap. By a railroad technique, the advancement flap was dragged below the hammock flap upwards. This “bridge” flap was split into anterior and posterior lamina. Posterior lamina consisted of conjunctiva and capsulopalpebral ligament. Anterior lamina consisted of skin and orbicularis oculi. Upper eyelid was also divided into anterior and posterior laminae.

**Fig. 3:**
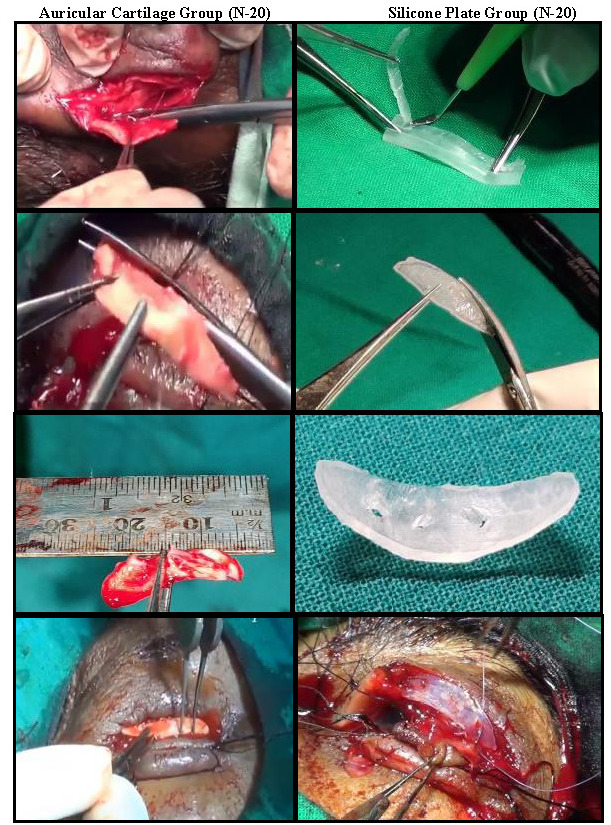
The harvested autogenous auricular cartilage and silicone plate fashioned from 279 retinal buckle. This was placed over the posterior lamellar bay in two separate patients

Posterior lamina is made of conjunctiva and aponeurosis of LPS muscle and orbital septum. Anterior lamina is made of skin, and orbicularis oculi muscle only. Both the posterior laminae of the bridge flap and the upper lid were sutured to make the posterior laminar “bay” for placing the auricular cartilage or silicone plate. The silicone plate/ auricular cartilage was positioned over the posterior lamina and secured in place by 5-0 polyglactin suture (**Fig. 3**). Anterior myo-cutaneous flap was advanced and positioned. The lid defect was sutured, sandwiching the silicone plate/ auricular cartilage between the two laminae. This bridge flap was maintained for the next six weeks. In Stage II of the Modified Cutler–Beard procedure, an incision with convexity downwards was made to separate the bridge flap. Double-armed 6-0 polyglactin sutures were used to reform newly-made lid margin and the knot was placed over the skin surface. In this manner, the upper eyelid was reconstructed.

## Post-operative evaluation

Patients were examined and photographed at standard postoperative visits at one week, one month and six months against the following parameters: post-operative LPS action, post-operative marginal reflex distance, post-operative eye lid closure, post-operative palpebral fissure height, postoperative lid contour and cosmesis, post-operative central lid thickness, examination of the cornea, histopathological reports, post-operative MRI orbit, and clinical photographs (**Fig. 1**, **2**, **Table 1**, **2**).

## Material description: silicone plate

**Fig. 4:**
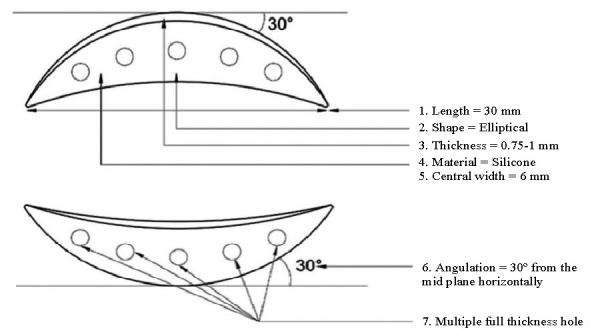
Schematic diagram of silicone plate with specification

The silicone plate was made inside the operation theatre under strict aseptic measures (**Fig. 3**, **Fig. 4**). It was then packed and sent for sterilization with ethylene oxide [**3**].

## Harvesting auricular cartilage

On the ear ipsilateral to the eyelid with the defect, a vertical incision was made at the medial side of the pinna and adequate tissue dissection was done to expose the cartilage (**Fig. 3**) [**2**]. A wedge of cartilage, measured appropriately to replace the upper lid tarsus, was dissected and removed. Interrupted 5-0 black silk suture was used to close the wound. Loose tissue was scraped off from its surface to make it thinner and more even. The autogenous auricular cartilage had multiple irregularities on its surface and was flattened, which was responsible for the uneven lid contour and margin irregularities in the early post-operative period. A 5-0 polyglactin suture was used to secure the cartilage graft over the posterior lamellar bay.

## Results and analysis

The statistical data analysis was done using IBM SPSS statistics version 25.0. As the data was non-normal in distribution, non-parametric tests (Mann-Whitney U test) were employed to find out any statistically significant difference between the two groups. 

The mean age with the standard deviation was 55.5 ± 9.88 years in the silicone plate group and 58.5 ± 8.65 years in the autogenous auricular cartilage group.

The female: male ratio in the silicone plate group and autogenous auricular cartilage group was 1.2:1.0 and 1.5:1.0, respectively.

The range of the created defect size ranged from 70-100% in both groups. The mean percentage of intraoperative upper lid defect created was 85.45 ± 9.8% in the silicone plate group and 86.62 ± 10.31% in the autogenous auricular cartilage group. The median value was 86.5% and 86% in both groups, respectively.

The mean time taken for stage one of the Modified Cutler Beard procedure in the silicone plate group was 47.5 ± 5.96 minutes, whereas the mean time taken for the same in the autogenous auricular cartilage group was 168.5 ± 12.36 minutes.

**Fig. 5:**
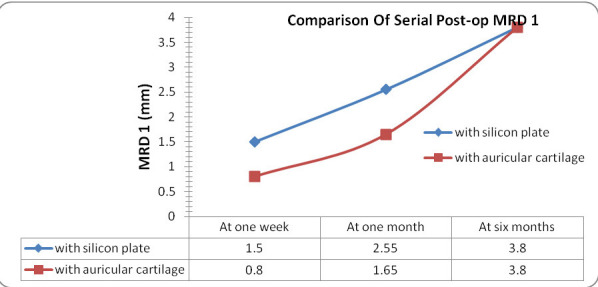
Comparison of serial post-operative MRD

The mean pre-operative MRD 1 in the silicone plate group and autogenous auricular cartilage group was -2.95 ± 1.19 mm and -3.05 ± 1.05 mm, respectively. The mean post-operative MRD 1 for the silicone plate group at one week, one month and six months post-operative period was 1.5 ± 0.6 mm, 2.55 ± 0.5 mm and 3.8 ± 0.4 mm, respectively, whereas the corresponding values for the auricular cartilage group were 0.8 ± 0.4 mm, 1.65 ± 0.46 mm, and 3.8 ± 0.41 mm, respectively (**Fig. 5**).

**Fig. 6:**
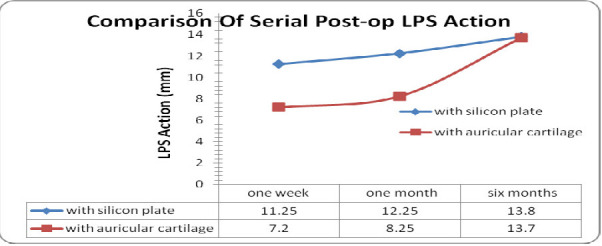
Comparison of serial post-operative LPS Action

The mean pre-operative LPS action in the silicone plate group and autogenous auricular cartilage group was 1.2 ± 1.1 mm and 1.0 ± 0.9 mm, respectively. The mean post-operative LPS action for the silicone plate group at one week, one month and six months post-operative period was 11.25 ± 0.6 mm, 12.25 ± 0.9 mm and 13.8 ± 0.4 mm, respectively, whereas the corresponding values for the auricular cartilage group were 7.2 ± 0.7 mm, 8.25 ± 0.7 mm, and 13.7 ± 0.4 mm, respectively (**Fig. 6**).

The mean pre-operative Palpebral Fissure Height (PFH) in the silicone plate group and autogenous auricular cartilage group was 2.05 ± 1.19 mm and 1.95±1.05 mm respectively.

**Fig. 7:**
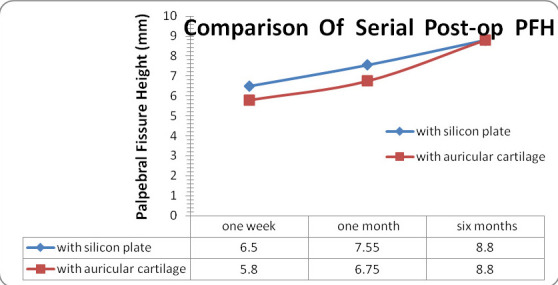
Comparison of serial of post-operative PFH

The mean post-operative PFH for the silicone plate group at one week, one month and six months post-operative period was 6.5 ± 0.60 mm, 7.55 ± 0.51 mm and 8.8 ± 0.41 mm, respectively, whereas the corresponding values for the auricular cartilage group were 5.8 ± 0.41 mm, 6.75 ± 0.41 and 8.8 ± 0.41 mm, respectively (**Fig. 7**). 

The mean post-operative lid thickness for the silicone plate group at one week, one month and six months post-operatively was 6.6 ± 0.50 mm, 5.8 ± 0.66 mm and 4.4 ± 0.17 mm, respectively, whereas the corresponding values for the auricular cartilage group were 7.4 ± 0.27 mm, 6.8 ± 0.34 mm, and 4.4 ± 0.08 mm, respectively. The calculated “U” value (Mann-Whitney test) at one week and one month post-operative period shows a statistically significant difference between the two groups for MRD 1, LPS action, palpebral fissure height and central lid thickness, which became statistically insignificant at six months.

## Lid contour

The lid contour was assessed with the help of clinical photographs taken at each post-operative visit. The assessment of anterior and posterior border of upper eyelid margin, central and peripheral arch of upper eyelid margin, lateral and medial canthi was done and compared with the opposite eye in all the patients. Synchronized blinking action was evaluated and lid lag, lid deformity and ptosis if any, was looked for. For the silicone plate group, the lid contour was found to be maintained in a larger number of patients at each follow-up than in the autogenous auricular cartilage group (**Table 3**).

**Table 3:** Number of patients with maintained lid contour at each post-operative visit in both groups

Number of patients with maintained lid contour	Silicone plate group	Autogenous auricular cartilage group
At one week	14	11
At one month	16	14
At six months	20	16

## Discussion

In this study, we discussed the comparison of using novel silicone plate vs. ear cartilage for the upper lid reconstruction after resection of large upper lid tumor. In 2007, DeSousa JL et al. conducted a study in Australia, in which 5 patients underwent lid reconstruction surgery after the excision of tumor; the posterior lamella was repaired with composite graft and anterior lamella by skin-muscle flaps [**12**]. With this procedure, they encountered major complications like lagophthalmos, ptosis, lid retraction, and ectropion. Post-operatively, the lids were stiff and immobile and required revision surgery. Thus, the total eyelid defects required a careful pre-planning and vascularized flaps were preferred over grafts in these cases.

For Koshima I et al., who used an ear helix flap for the reconstruction of a large upper eyelid defect, establishing proper venous drainage was a major challenge [**13**]. In case of massive facial burn, normal facial tissue for graft is scarce, so free dorsal pedicle flap with septal cartilage support has been used in 1999, in the USA, by KN et al., for the reconstruction of the lid [**14**]. In 2018, Qingji Li performed a two-stage lamellar rotation surgery for the large upper eyelid defect (about 75%), but the main drawback was a complex time-consuming procedure and decrease in length of the horizontal palpebral fissure [**15**]. Kersten et al. used tarsal rotation flaps for the upper eyelid reconstruction [**16**]. 

The modifications of the Cutler Beard procedure have been made in the past, in which various materials have been used as tarsal plate substitutes [**17**], like donor sclera [**18**,**19**], Achilles tendon [**20**], mucoperiosteal hard palate graft [**21**] and auricular cartilage, harvested from the other surgical sites of the body, or cadaver. So, there was a risk of post-operative complications at the second surgical site or disease transmission from donor to recipient. In 2016, Mandal SK et al. used autogenous auricular cartilage as an alternate material to tarsal plate in 16 patients with malignant upper eyelid tumors following excision for lid reconstruction [**10**]. There was a significant improvement in the post-operative parameters like MRD1, LPS action in all the patients, offering acceptable functional and aesthetic outcome. The limitation was a second surgical site, uneven thickness and irregular surface of the collected auricular cartilage, whose curvature did not match with that of the healthy lid. Despite remarkable post-operative tissue acceptance and adaptability, the newly made upper lid was thicker, thus cosmetically fair to poor in appearance. Moreover, it was a time consuming procedure. The harvesting of the ear cartilage required trained and skilled assistance, occasional cartilage related complications like breakage, or ear skin perforation might occur. Hence, the search for ideal tarsal plate substitute continued. In a very recent study, again by Mandal SK et al. (2021), a novel tarsal plate substitute in the form of a silicone plate was proposed and the cosmetic and functional outcome was studied [**11**]. The elliptical design of a silicone plate was custom made to resemble the tarsal plate in its dimensions. The second stage surgery was performed after six weeks and both lids were separated. Thus, the silicone plate remained in situ for the rest of the patient’s life, restoring functionality with the patient blinking more than 1000 times per day. The major advantage of using silicone plate over auricular cartilage was the absence of a second surgical site and a much shorter operating time.

In this study, the post-operative MRD-1 and LPS action values in the silicone plate group was better than in the group using ear cartilage in the 1st week and 1st month post-operative follow-up, which was statistically significant, but at the 6th month follow-up, the difference in values in the two groups was not statistically insignificant. The mean post-operative MRD 1, LPS action values obtained for both groups at one week, one month and six months post-operative period were similar to those obtained by Mandal SK et al. in 2016 and 2021 [**10**,**11**]. 

The post-operative PFH values for the silicone plate group at 1st week and 1st month post-operative follow-up was better than in the auricular cartilage group, which was statistically significant, but at the 6th month follow-up, the difference in values in the two groups was not statistically insignificant.

The initial post-operative period of the lid thickness in the auricular cartilage group was longer than that in the silicone plate group, it became comparable and almost equal in the late post-operative period, i.e., at six months. Unusual hypertrophy of the newly made upper lid was noted in two patients of the auricular cartilage group and one patient of the silicone plate group, which subsequently normalized at six months post-operative period and did not require any surgical intervention. Two patients belonging to the silicone plate group showed decreased thickness of eyelid at six months. In stage II upper lid reconstruction, incision was made a little beyond the inferior margin of the tarsal plate substitute to prevent its extrusion. A possible explanation for this was: division of the hammock flap in stage II of the modified Cutler Beard procedure, was done further down, away from the silicone plate positioned during stage I and hence, the lower margin of the tarsal substitute was not adjacent to the newly formed lid margin. 

The upper eyelid lid contour in the silicone plate group, at one week, one month and six months post-operative period, was well maintained in 14, 16 and 20 patients, respectively as opposed to the autogenous auricular cartilage group in which the corresponding values were 11, 14 and 16, respectively. The scleral buckle, out of which the silicone plate was made, had a smooth surface and intrinsic curvature, which in turn imparted an angulation of 30° from the mid plane to the silicone plate. That curvature matched that of a healthy upper eyelid. On the other hand, the harvested auricular had an irregular surface and was of uneven thickness throughout its length. Moreover, its curvature did not match with that of the lid. So, despite excellent post-operative tissue acceptance and tissue adaptability, the contour of the newly made eyelid was cosmetically not up to the mark. One patient in the silicone plate group was noted to have conjunctival overgrowth at the upper eyelid margin at one month follow up, which was trimmed and lid margin anatomy was restored at six months follow-up. A possible explanation was that at the time of division of the advancement flap during the second stage incision with convexity downward, the incision went tangentially more towards the conjunctival surface such that the overhanging conjunctiva was preserved more than necessary. 

One patient in the silicone plate group was found to develop upper lid grade 1 entropion at first month post-operative follow-up. This was probably due to a vertical rather than a tangential division of the flap during the second stage surgery. The post-operative oedema caused the lid to turn inward. Later, when the oedema subsided, the lid margin returned to its normal position, thus correcting the entropion. Another patient in the autogenous auricular cartilage group was found to develop lower lid ectropion after one month of the second stage. This was an elderly patient with pre-existing lid laxity. Both the complications resolved spontaneously by six months post-operative period with proper eyelid massage, and did not require any surgical intervention.

One patient in the silicone plate group developed transient lagophthalmos at first week postoperative visit. The lagophthalmos appeared only when the patient was actively fixating on a target on command and disappeared when the patient was inattentive or distracted. This transient lagophthalmos disappeared by one month post-operative period. 

The silicone plate had extruded in two cases within one month of stage II surgery. Infection of the surgical site along with poorly controlled diabetes was the cause in one patient. The other patient suffered an implant extrusion due to a wrongly fashioned silicone plate, which was thicker and had sharp angles implanted on thin posterior lamella. Both these patients underwent near total replacement, hence despite implant extrusion, their long-term post-operative parameters of MRD 1 and LPS action remained within acceptable limits (3 mm and 12 mm, respectively). Neither of the patients developed lid shrinkage, entropion or ectropion.

Hawes et al. discussed the complications encountered in patients whose upper eyelids have been reconstructed using tarsoconjunctival grafts [**22**,**23**]. Major complications included upper lid retraction, wound dehiscence, cicatricial ectropion and lower lid laxity. Minor complications included notching of the donor or recipient lid margin. Also, composite grafts to the eyelid resulted in lid contraction, discoloration, poor healing and immobility in quite a few cases. No such problems were not encountered in our study in either group.

Corneal complications like abrasion or foreign body sensation were not noted in any patient from either group in our study. This was because, for both the silicone plate and ear cartilage, the tarsal substitute was ensheathed by newly made fibrous capsule. This was formed by anterior or posterior lamella. The multiple holes in the silicone plate allowed fibrovascular proliferation, which prevented its migration and thus, helped preserving corneal health. The lid margin was covered by the overhanging conjunctiva making it smooth, which helped maintain the tear film stability and corneal health. The lid margin was formed with 6-0 double armed polyglactin sutures such that the knots remained on the skin surface, which helped prevent suture related corneal complications.

The average operating time of stage one of the Modified Cutler Beard procedure in case of the silicone plate group was 47.5 ± 5.96 minutes, whereas the same took an average of 168.5 ± 12.36 minutes in the autogenous auricular cartilage group (p<.001). Although the site of the autogenous cartilage harvesting has a cosmetic advantage, donor site related complication is not unheard of.

The use of synthetic tarsal plate substitutes like the bioengineered TarSys™ [**24**] and MEDPOR® have been described in literature, but necessary graft removal of foreign body giant cell reaction to TarSys™ has also been reported in two occasions [**25**,**26**]. MEDPOR® sheet has been used as an upper eyelid tarsal plate substitute in a study conducted in Korea in 2009 [**27**]. The outcome was quite satisfactory, except for an early postoperative lid stiffness that resolved spontaneously. However, the main issue of concern is the cost factor and availability. The cost of bioengineered TarSys™ is $385, donor sclera approximately $300 to $600 and Tendoachilles grafts were nearly about $700 to $1100 [**20**]. Although allogenic and synthetic tarsal substitutes are ready to use and thereby largely reduce the surgical time, their cost and availability limit their use in developing countries [**28**]. These drawbacks with the use of allogenic or autogenous grafts have been overcome with the use of a novel synthetic silicone plate. The cost of the novel silicone plate is around 483 INR (6.5$) [**11**]. The silicone plate is cost effective, and this has enabled its use in socioeconomically weaker populations. Thus, though the surgical outcomes of use of autogenous auricular cartilage and silicone plate are comparable, silicone plate, as a tarsal substitute, is logistically a much better option. 

## Conclusion

Our study showed that, although the surgical outcome in terms of functionality in both groups of patients were similar, the cosmetic outcome in terms of lid contour maintenance appeared to be better in the silicone plate group. Besides, with the use of pre-made silicone plate, surgical time was markedly reduced. Harvesting autogenous auricular cartilage from the back of the pinna on the other hand is a time consuming and skilled procedure, involving a second surgical site, which increases post-operative morbidity and chances of donor site related complications. Additional care of the second surgical site is required in the post-operative period. The silicone plate is widely available and cost effective. Moreover, there are no concerns about communicable disease transmission like HIV or hepatitis and hence eliminates cost of donor screening. The silicone plate is an inert, ultra-thin, light weight, tissue and time tested, synthetic material with a smooth surface and an intrinsic curvature that helps maintain the upper eyelid architecture and margin contour better than cartilage group. Autogenous auricular cartilage on the other hand, is flat and has an irregular surface and thicker than silicone plate, thus provides a less optimal lid contour and aesthetic appearance. Though both autogenous auricular cartilage and silicone plate are safe and cost-effective tarsal substitutes, providing satisfactory functional and cosmetic results, silicone plate is a more promising alternative. Although longer follow-ups are required to gain a better understanding of long-term outcomes, silicone plate is reckoned to become the next generation material of choice as tarsal substitute in the future. 

**Conflict of Interest statement**

The authors state no conflict of interest.

**Informed Consent and Human and Animal Rights statement**

Informed consent has been obtained from all individuals included in this study.

**Authorization for the use of human subjects**

Ethical approval: The research related to human use complies with all the relevant national regulations, institutional policies, is in accordance with the tenets of the Helsinki Declaration, and has been approved by the review board of Department of Orbit, Oculoplasty and Reconstructive Surgery, Regional Institute of Ophthalmology, Kolkata, India.

**Acknowledgement**

I acknowledge Dr. Hays T. Cape, Dr. Fabliha Anbar Mukit, Dr. Brian T. Fowler, and Professor James C. Fleming, who have presented this paper in ARVO, in 2023, in New Orleans, LA, and my co-author who has presented this paper in All India Ophthalmological Conference, in 2022, in Mumbai.

**Sources of Funding**

None.

**Disclosures**

None.
